# Enhanced discriminative aversive learning and amygdala responsivity in 5-HT transporter mutant mice

**DOI:** 10.1038/s41398-019-0476-8

**Published:** 2019-04-17

**Authors:** João Lima, Trevor Sharp, David M. Bannerman, Stephen B. McHugh

**Affiliations:** 1 Department of Experimental Psychology, University of Oxford, Oxford, UK; 2 Danish Research Institute of Translational Neuroscience (DANDRITE), Department of Molecular Biology and Genetics, Aarhus University, Aarhus, Denmark; 3 Department of Pharmacology, University of Oxford, Oxford, UK; 4 Medical Research Council Brain Network Dynamics Unit at the University of Oxford, Oxford, UK

**Keywords:** Neuroscience, Physiology

## Abstract

Genetic variation in the human serotonin transporter (5-HTT) has been linked to altered fear learning but the data are inconsistent and the mechanism is unclear. The present study investigated conditioned aversive learning in 5-HTT knockout (KO) mice while simultaneously recording neural network activity (theta oscillations) and hemodynamic responses (tissue oxygen delivery) from the amygdala, a brain region necessary for forming fearful memories. Conditioned aversive learning was measured using a discrimination learning task in which one auditory cue was paired with foot-shock, whereas a second auditory cue was not. Compared with wild-type mice, 5-HTTKO mice exhibited faster discrimination learning. This effect was associated with stronger theta frequency oscillations and greater hemodynamic changes in the amygdala in response to both the emotionally relevant cues and the unconditioned foot-shock stimulus. Furthermore, hemodynamic responses to the unconditioned stimulus predicted behavioral discrimination performance the following day. Acute pharmacological 5-HTT blockade in wild-type mice produced a similar effect, to the extent that administration of citalopram during the fear conditioning sessions enhanced fear memory recall. Collectively, our data argue that loss of 5-HTT function enhances amygdala responsivity to aversive events and facilitates learning for emotionally relevant cues.

## Introduction

The serotonin transporter (5-HTT) regulates serotonin (5-HT) availability at the synapse, and is the target of selective serotonin reuptake inhibitors (SSRIs), which are currently the mainline treatment for depression and anxiety disorders. A substantial research effort has examined the influence of variation in the human 5-HTT gene on the risk for developing anxiety and depression, as well as other aspects of emotional function. These studies have focused on the 5-HTT gene-linked polymorphic region (5-HTTLPR), which is an insertion/deletion polymorphic site that generates long and short alleles. The latter allele is linked to reduced 5-HTT expression (relative to the long allele, specifically the L_A_ subtype), which is thought to predispose individuals to higher neuroticism (an anxiety-related personality trait), and increased risk of depression and anxiety, especially when combined with aversive life experiences^1–5^. Moreover, the risk of psychiatric disorders associated with reduced 5-HTT expression is thought to be underpinned by elevated amygdala responsivity to fearful stimuli as detected in functional Magnetic Resonance Imaging (fMRI) Blood Oxygen Level Dependent (BOLD) studies^4,5^.

However, collectively these findings remain controversial, with large sample studies and meta-analyses being inconsistent in their outcome^6–10^. Interpretation is further complicated by the difficulty establishing whether 5-HTTLPR genotype influences 5-HTT expression (and thereby synaptic 5-HT availability) in human brain in vivo^11,12^. Nevertheless, unraveling associations between 5-HTT expression and anxiety/fear is highly relevant in the context of recent theories proposing that altered 5-HT availability modulates environmental sensitivity through altering the plasticity of synapses involved in emotional learning^13–17^.

In comparison with human studies, animal experiments demonstrate more reliable evidence that altered 5-HTT expression influences anxiety. In particular, variation in the 5-HTT has been modeled in mice by both 5-HTT knockout (5-HTTKO) and 5-HTT overexpression (5-HTTOE), which demonstrate elevated and reduced extracellular 5-HT levels, respectively^18^. Importantly, and as predicted by human 5-HTT gene association studies (see above), 5-HTTKO mice exhibit greater anxiety in unconditioned tasks such as the elevated plus maze and novelty suppressed feeding, whereas 5-HTTOE mice exhibit reduced anxiety in these tasks^19–21^.

The influence of altered 5-HTT expression on conditioned aversive learning has also been investigated in rodents although the data are less clear. Thus, 5-HTTKO mice and rats are reported to exhibit normal fear learning^22–24^ rather than increased fear learning that human data and the above 5-HT emotional plasticity theory predict. However, these 5-HTTKO rodent studies used “single-cue” conditioning paradigms (i.e., several exposures to one auditory cue paired with foot-shock); and since wild-type (WT) animals typically display high levels of fear-related behavior (“freezing”) in such paradigms, it is difficult to detect further increases.

In contrast, 5-HTTOE mice exhibit impaired fear learning^25,26^. Moreover, 5-HTTOE mice exhibit reduced amygdala tissue oxygen (T_O2_) responses to aversive cues as measured by voltammetry, as well as reduced cue-evoked theta oscillations in the amygdala local field potential (LFP)^25^. Voltammetric T_O2_ measurements are considered a hemodynamic parameter that reflects on-going neuronal activity, analogous to the BOLD signal in fMRI^25,27,28^. Moreover, theta oscillations in the amygdala are thought to be a neurophysiological substrate for the acquisition of fearful memories^27,29^.

To clarify the impact of 5-HTT loss on emotional learning, the present study investigated conditioned aversive learning and amygdala activity in 5-HTTKO mice using a discriminative fear learning paradigm rather than the “single-cue” fear paradigm used in previous investigations of 5-HTTKO rodents. Mice were trained to discriminate between two distinct auditory stimuli, one that was paired with foot-shock (CS+) and another that was never paired with foot-shock (CS–). In a subset of mice, hemodynamic responses and LFPs were simultaneously recorded from the amygdala during different stages of fear learning. An additional experiment tested the effect of acute pharmacological 5-HTT blockade on aversive learning in WT mice because of reports that the anxiety phenotype of 5-HTTKO mice is of developmental origin rather than an on-going loss of 5-HTT function^30,31^.

## Materials and methods

Detailed methods can be found in the Supplementary Material.

### Subjects

Experiment 1 (study of 5-HTTKO mice) used 31 WT mice (15 males and 16 females) and 29 5-HTTKO mice (15 males and 14 females). Mice were generated as described previously^32^ and backcrossed onto a C57BL/6J background for at least eight generations. Experiment 2 (study of 5-HTT blockade) used 96 female C57BL/6J mice (Charles River Laboratories, Kent, UK). Mice were 4–10 months (5-HTTKO and WT) or ~2 months old (C57BL/6J) at the start of testing. All experiments were conducted in accordance with the United Kingdom Animals Scientific Procedures Act (1986) under project licenses PPL 30/2561 and 30/3068, and were approved by local ethical review for the University of Oxford.

### Surgery

For Experiment 1, a subset of mice (WT 8 females and 6 males: 5-HTTKO 7 females and 7 males) were stereotaxically implanted (under isoflurane anesthesia) with electrodes into the basolateral amygdala (BLA) to record T_O2_ and LFPs. Co-ordinates were −1.35 mm anterior/posterior, ±3.15 mm medial/lateral and −5.00 mm dorsal/ventral, relative to bregma (Supplementary Fig. S1).

### Amygdala T_O2_ and LFP recordings

Signals for T_O2_ were measured using constant potential amperometry, as described in detail previously^28,33^. A constant potential (−650 mV relative to a reference electrode) was applied to the electrode, which results in the electrochemical reduction of O_2_ on the electrode surface such that local changes in O_2_ concentration produce directly proportional changes in the measured Faradaic current. LFPs were recorded using a differential amplifier (DP-301, Warner Instruments, CT, USA) and sampled continuously at 4 kHz.

### Fear conditioning

**Fig. 1:**
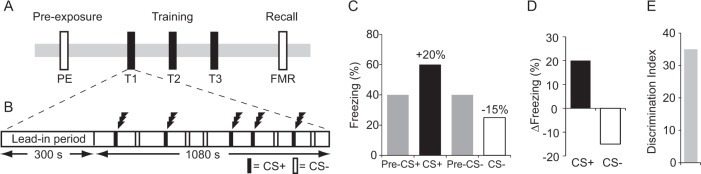
Experimental design. **a** Schematic of the training paradigm. **b** Each session contained a 300-s lead-in period followed by five CS+ and five CS– presentations, pseudo-randomly interleaved, and with a different presentation sequence on each day. On training days (T1–T3), the CS+ co-terminated with foot-shock (lightening icon), whereas the CS– was never paired with foot-shock. No shocks were given during pre-exposure (PE) or fear memory recall (FMR) sessions. **c, d** Illustration of how percentage freezing scores during pre-CS and CS periods (shown in **c**) were used to create Δfreezing difference scores [CS minus pre-CS] (shown in **d**). **e** A discrimination index was generated by subtracting CS– from CS+ Δfreezing scores (e.g., +20 – (–15) = +35)

Fear conditioning was conducted in one of two operant chambers (ENV-307A, Med Associates Inc., IN, USA), each with distinct visual and olfactory cues. Mice (total of 60 including 28 with implanted BLA electrodes) underwent a pre-exposure day, three training days (T1, T2, and T3), and a fear memory recall day (FMR; Fig. 1a). Training was performed in one context (e.g., context A) and recall/extinction was performed in a different context (e.g., context B if trained in context A). Contexts were counterbalanced across mice.

Mice were trained to discriminate between two distinct auditory cues (tone and white noise), with one conditioned stimulus (e.g., CS+ = tone) always paired with foot-shock during the training sessions and the other never paired with shock (e.g., CS– = white noise). Allocation of tone and white noise to the CS+/CS– was fully counterbalanced across mice. During each session, mice were presented with 10 auditory cues (5 × 2900 Hz tone, 5 × white noise; all 72 dB and 30-s duration) in a pseudo-randomly interleaved order with a mean inter-cue interval of 80 s (range 60–100 s). On training days, each of the five CS+ trials co-terminated with mild foot-shock (0.3 mA, 0.5 s; Fig. 1b). No shocks were given during the pre-exposure or recall sessions.

### Data analyses

#### Behavior

Freezing responses were assessed by automated movement detection using software running in NIH image^34^. To calculate cue-evoked freezing responses (Δfreezing), % freezing in the 30 s before cue onset was subtracted from % freezing during cue presentation (Fig. 1c, d). Cue-evoked freezing greater than the pre-cue baseline yields positive scores and cue-evoked freezing less than pre-cue period yields negative scores (Fig. 1c, d). A discrimination index was calculated by subtracting the Δfreezing evoked by the CS– from the Δfreezing evoked by the CS+ (Fig. 1e). Note that there were no genotypic differences in freezing during pre-cue or cue periods during the pre-exposure session (all *F*-values < 1.4, all *p*-values > 0.2; Supplementary Fig. S2). Also freezing levels during the pre-cue periods did not differ between genotypes during training or recall (no main effect of genotype or genotype × day interaction: *F* < 1, *p* > 0.8). Freezing responses during pre-cue and cue periods for all days are shown in Supplementary Fig. S3.

#### Cue-evoked TO_2_ responses

Cue-evoked T_O2_ responses were calculated by subtracting the mean T_O2_ signal during the 5 s before cue onset (i.e., pre-cue baseline) from the T_O2_ signal during the 30-s cue presentation. Then, the 30-s signal was divided into 15 2-s time bins (i.e., 0–2 s, 2–4 s, 4–6 s…28–30 s) with each data point equal to the mean value during each 2-s time bin. The foot-shock (US) was 0.5-s duration, and US-evoked signals were measured in the 30 s after the shock (excluding 0.5 s of US administration). T_O2_ responses were averaged over the five CS+, CS–, and US trials of each session. In addition, a regression analysis was used to investigate whether T_O2_ signals predicted behavioral discrimination. For this analysis, the maximum T_O2_ signal (i.e., the peak value in one of the 15 time bins) was calculated during the CS+, CS–, and US periods. The regression model used these values (T_O2__CS+, T_O2__CS–, and T_O2__shock) on training days T1, T2, and T3 to predict behavioral discrimination on subsequent training days T2 and T3 and the FMR session, respectively. Stepwise linear regression was used to determine the maximum variance explained with the fewest independent variables.

#### LFPs

LFPs were band-pass filtered between 1 and 80 Hz. Power spectra were calculated during the 10 s after cue onset for each trial and then averaged over the five CS+ or five CS– trials in each session. Spectra were computed in MATLAB (The Mathworks, MA, USA). For statistical analysis, the power spectral density in each frequency bin (Φ_i_,) was transformed into a proportion of the total power between 1 and 40 Hz:

$$P_i = \left( {\frac{{\Phi _i}}{{\mathop {\Sigma }\limits_1^{40} \Phi }}} \right),$$

where *P*_i_ = proportional power, Φ_i_ = raw power density (mV^2^/Hz).

From these proportional spectra, we determined the peak power in the theta frequency range (5–10 Hz).

#### Histology

At the end of the experiments, mice implanted with electrodes were injected with sodium pentobarbitone; 200 mg/kg) and perfused transcardially with physiological saline followed by either 10% formol saline or 4% paraformaldehyde. Coronal sections (40 μm) were stained with cresyl violet to determine that electrodes were in the BLA (Supplementary Fig. S1).

#### Drug treatment

In Experiment 2, a separate group of mice (*n* = 96) were injected with saline or citalopram (10 mg/kg, i.p.; Tocris, UK) 30 min before each training and/or recall session using a fully counterbalanced design (Table 1). The behavioral paradigm was identical to that used in Experiment 1, except there was no pre-exposure session and only 2 days of training. There were four separate treatment groups: mice given saline during training and recall (Sal_Sal), mice given citalopram during training and saline during recall (Cit_Sal), mice given saline during training and citalopram during recall (Sal_Cit), and mice given citalopram during both training and recall (Cit_Cit).

**Table 1:** Experimental design for the citalopram study

	Training day 1	Training day 2	Recall
Sal_Sal (*n* = 24)	Sal	Sal	Sal
Sal_Cit (*n* = 24)	Sal	Sal	Cit
Cit_Sal (*n* = 24)	Cit	Cit	Sal
Cit_Cit (*n* = 24)	Cit	Cit	Cit

Mice (*n* = 96) were divided into four groups and injected with either saline (Sal) or citalopram (Cit; 10 mg/kg, i.p.) 30 min before each training or recall session

#### Statistical procedures

Data were analyzed using analysis of variance (ANOVA) in SPSS (version 22, IBM, USA). ANOVAs are described in the form: A_2_ × B_3_, where A is a factor with two levels and B a factor with three levels. All graphs show the mean ± 1 standard error of the mean (SEM). The family-wise error was set at *α* = 0.05, with all tests performed as two tailed, and the Dunn–Sidak method employed to correct for multiple comparisons.

## Results

### Facilitated discriminative aversive learning in 5-HTTKO mice

**Fig. 2:**
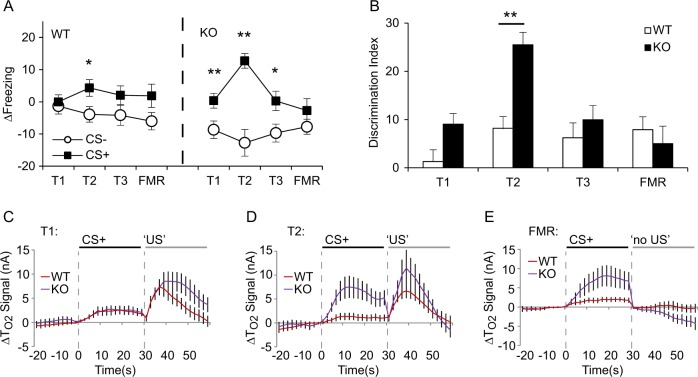
Behavioral and amygdala hemodynamic tissue oxygen responses (ΔT_O2_ signals) during discriminative aversive learning in wild-type (WT) and 5-HTTKO (KO) mice. **a** 5-HTTKO mice exhibited larger differences between CS+ and CS– evoked Δfreezing scores during training. Note that positive Δfreezing scores indicate an increase in freezing compared with the pre-CS period whereas negative scores indicate a decrease in freezing compared with the pre-CS period. **b** The discrimination index (CS+Δfreezing – CS– Δfreezing scores) was significantly higher in 5-HTTKO than WT mice during T2. **c–e** Mean amygdala hemodynamic responses before, during, and after CS+ presentation in WT and 5-HTTKO mice on T1, T2, and fear memory recall (FMR). Note that T_O2_ signals were higher in 5-HTTKO mice following foot-shock on T1 and higher during CS+ presentations on T2. **a**, **b** Mean ± SEM, *n* = 60 mice. **c**–**e** Mean ± SEM, *n* = 22–24 mice. *p < 0.05, **p < 0.01

Both genotypes learned to discriminate between the aversive cue (CS+) and non-aversive cue (CS–) by training day 2 (T2) but this discrimination was markedly stronger in 5-HTTKO mice (Fig. 2a, b). Analysis of Δfreezing scores (ANOVA model: genotype_2_ × sex_2_ × day_2_ × CS type_2_ × trial_5_, *n* = 60 mice) revealed greater freezing evoked by the CS+ than CS– (main effect of CS type: *F*(1, 56) = 23.4, *p* < 0.001) and superior discrimination in 5-HTTKO compared with WT mice (genotype × CS type interaction: *F*(1, 56) = 7.6, *p* = 0.01). Moreover, 5-HTTKO mice exhibited significant discrimination during training day 1 (T1), whereas WT mice did not (*p* = 0.01 versus *p* = 0.7; see Supplementary Fig. S3). In addition, analysis of the discrimination index (calculated as [CS+] – [CS–] Δfreezing scores), revealed significantly stronger discrimination in 5-HTTKO versus WT mice during T2 (*F*(1, 56) = 8.0, *p* = 0.006; Fig. 2b), thus 5-HTTKO mice exhibited better discriminative aversive learning. However, with one additional training day (T3), discrimination now did not differ between WT and 5-HTTKO mice, and there was no effect of genotype or genotype × CS type interaction during T3 or during FMR (*F* < 2.5, *p* > 0.1; see Fig. 2a, b).

### Stimulus-evoked amygdala T_O2_ responses are greater in 5-HTTKO mice

Voltammetric T_O2_ signals are considered hemodynamic responses, which reflect on-going neuronal activity, similar to the BOLD signal in fMRI, and in the amygdala provide a neural correlate of aversive learning^27^. During the pre-exposure session, T_O2_ responses did not differ between genotypes (no effect of genotype, stimulus type or interaction: *F* < 1.5, *p* > 0.2; Supplementary Fig. S2).

However, during fear learning, amygdala T_O2_ responses were greater in 5-HTTKO than WT mice. Analysis of T_O2_ signals during the first 2 training days (ANOVA: genotype_2_ × stimulus type_3 (CS–, CS+, US)_, × day_2_ × time bin_15_, *n* = 24 mice) revealed that during T1, shock-evoked T_O2_ responses were larger in 5-HTTKO mice (Fig. 2c). The higher amygdala T_O2_ signal in 5-HTTKO mice was not because the foot-shock evoked greater locomotor activity in 5-HTTKO versus WT mice (Supplementary Fig. S4). During T2, both CS+ evoked and CS– evoked T_O2_ responses were larger in 5-HTTKO versus WT mice (Fig. 2d; interactions between genotype, stimulus type and day: *F*(2, 44) = 3.6, *p* = 0.035, and genotype, stimulus type, day, and time bin: *F*(28, 616) = 2.4, *p* < 0.001; CS– responses are shown in Supplementary Fig. S5). Note that both genotypes had larger T_O2_ responses to the CS+ than the CS–, but this was not apparent in WT mice until training day T3 (Supplementary Fig. S5). Moreover, CS+ evoked amygdala T_O2_ responses were greater in 5-HTTKO compared with WT mice during FMR (main effect of genotype: *F*(1, 20) = 4.4, *p* = 0.049; genotype × CS type × time bin interaction: *F*(14, 280) = 2.2, *p* = 0.009), even though there were no genotype differences in behavioral responses on this day. Overall, cue-evoked amygdala T_O2_ responses were greater in 5-HTTKO than WT mice.

### Amygdala T_O2_ responses to foot-shock predict subsequent fear-related behavior

Multiple linear regression was used to investigate whether T_O2_ signals on day *n* predicted behavioral discrimination between the CS+ and CS– on day *n* + 1, as observed previously^27^. The dependent variable was the behavioral discrimination index and three independent variables were used: maximum T_O2_ signal during CS+ presentations (T_O2__CS+), maximum T_O2_ signal during CS– presentations (T_O2__CS–), and maximum T_O2_ signal in the 30 s following foot-shock (T_O2__shock). The optimum regression model contained only T_O2__shock and accounted for 7% of the variance in behavioral discrimination, which was significantly greater than zero (*F*(1, 68) = 5.1, *p* = 0.03, *R*^2^ = 0.07). There was a significant positive correlation between T_O2__shock and behavioral discrimination (standardized *β* coefficient = 0.27; *t*(68) = 2.4, *p* = 0.03).

Overall, and consistent with our previous report^27^, mice that exhibited larger T_O2_ responses to foot-shock exhibited stronger behavioral discrimination the following day. The larger shock-evoked responses in 5-HTTKO versus WT mice during T1 (Fig. 2c) may therefore explain, at least in part, the superior discrimination in 5-HTTKO mice during T2.

### Cue-evoked theta frequency neuronal oscillations are greater in 5-HTTKO mice

**Fig. 3:**
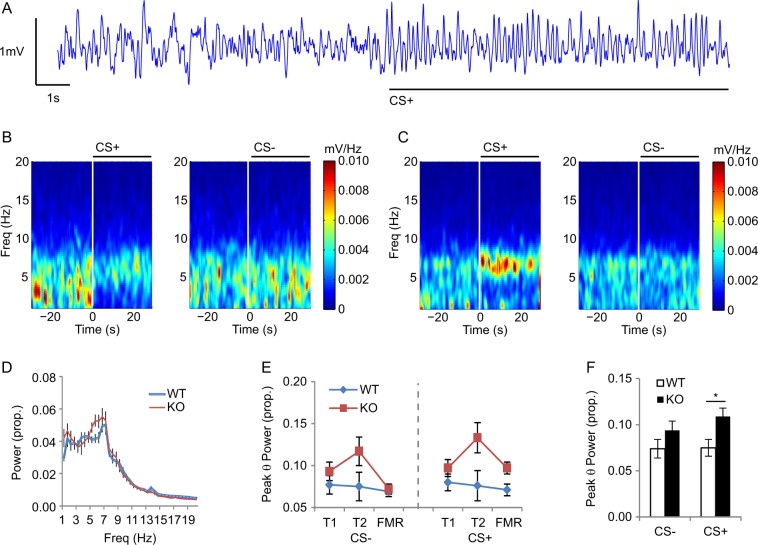
Cue-evoked neuronal oscillations in wild-type (WT) and 5-HTTKO (KO) mice during discriminative fear conditioning. **a** Raw local field potential trace showing theta activity evoked by CS+ onset. **b, c** Time-frequency spectrograms from one WT (**b**) and one 5-HTTKO (**c**) mouse during T2 illustrating how CS+ onset evoked a shift from delta-dominant (1–4 Hz) to theta-dominant (5–10 Hz) oscillations. **d, e** Peak CS+ evoked theta power was higher in 5-HTTKO than WT mice (**d**-**f**). Mean ± SEM, *n* = 24 mice. *p < 0.05

Next, experiments investigated whether the enhanced discrimination learning in 5-HTTKO mice was accompanied by changes in amygdala oscillatory neuronal activity. Previous work suggests that theta frequency (5–10 Hz) oscillations in the amygdala are a neurophysiological substrate of aversive learning^27,29,35^. Consistent with these previous reports, during training and FMR the onset of the CS+ increased theta oscillations and decreased delta oscillations (1–4 Hz). This increase in theta was evident in both genotypes but was stronger in 5-HTTKO than WT mice (Fig. 3).

Analysis of cue-evoked theta responses during T1, T2, and FMR (ANOVA: genotype_2_ × day_3_ × CS type_2_, *n* = 24 mice) revealed that theta oscillations were stronger during CS+ than CS– trials (main effect of CS type: *F*(1, 22) = 10.8, *p* = 0.003), and were stronger in 5-HTTKO than WT mice (genotype × CS type interaction: *F*(1, 22) = 7.1, *p* = 0.01). Pairwise comparisons revealed that CS+ evoked theta power was significantly greater in 5-HTTKO than WT mice (*p* = 0.02), with no genotypic difference for CS– trials (*p* = 0.15). Thus, 5-HTTKO mice exhibited enhanced amygdala theta oscillations during the aversive conditioned cues.

### 5-HTT blockade before training sessions facilitates subsequent FMR

5-HTTKO mice will experience loss of 5-HTT (and presumably greater synaptic 5-HT availability) throughout their entire lifetime. Hence, the facilitated fear discrimination in 5-HTTKO mice could be driven by loss of 5-HTT during development rather than adulthood^30,31^. To test whether the enhanced discrimination performance reflects loss of 5-HTT at the time of learning, WT mice were tested for discriminative fear conditioning, and administered citalopram (10 mg/kg, i.p.) or saline 30 min before each session in a fully counterbalanced design (see Table 1).

**Fig. 4:**
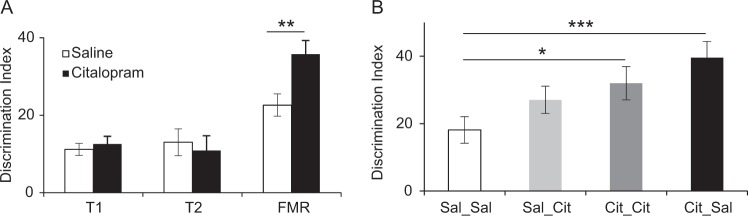
**Behavioral responses during discriminative aversive learning in saline- and citalopram-treated mice.** **a** Citalopram treatment 30 min before training sessions had no effect on CS+/CS– discrimination during the training sessions (T1, T2) but mice that received citalopram during training exhibited significantly better discrimination during the fear memory recall session (FMR). Note that during recall, the saline bar comprises the Sal_Sal and Sal_Cit groups and the citalopram bar comprises the Cit_Sal and Cit_Cit groups, so the effect was not due to citalopram on fear expression per se (*n* = 96 mice). **b** Discrimination during recall was strongest in those mice that received citalopram before training and saline before recall (Cit_Sal) and weakest in mice that received saline before both training and recall (Sal_Sal). ***p = 0.001, **p = 0.005, *p = 0.03

Analysis of the discrimination index revealed that citalopram treatment did not affect discrimination during the training days (no effect of day, drug treatment or interaction, *F* < 1, *p* > 0.5; Fig. 4). However, mice that received citalopram during training exhibited significantly better discrimination during FMR, regardless of whether they received saline or citalopram before the FMR session (main effect of treatment during training: *F*(1, 94) = 8.5, *p* = 0.005; Fig. 4a).

Comparison of the four treatment groups specifically during FMR (Fig. 4b) revealed that the Cit_Sal and the Cit_Cit groups exhibited significantly better discrimination than the Sal_Sal group (*p* = 0.001 and *p* = 0.03, respectively), and the Cit_Sal group discriminated better than the Sal_Cit group (*p* = 0.05). There were no other significant group differences. Citalopram did not affect freezing during pre-CS periods (Supplementary Fig. S6), nor responsivity to the foot-shock, except on the very first conditioning trial (Supplementary Fig. S7). Thus, mice that received citalopram during training exhibited enhanced FMR. These results suggest that 5-HTT blockade, and a likely increase in 5-HT availability at the time of conditioning, enhances aversive discrimination learning.

## Discussion

### Summary of results

The current study found that 5-HTTKO mice exhibited enhanced fear learning in a discriminative learning task in which one auditory cue (CS+) was paired with foot-shock, whereas a second auditory cue (CS–) was not. Importantly, it was found that this learning effect in 5-HTTKO mice was accompanied by larger amygdala tissue oxygen (T_O2_) responses to the conditioned stimuli (CS+, CS–), as well as the unconditioned stimulus (foot-shock). Tissue oxygen responses were predictive of subsequent learning, with larger shock-evoked responses predicting better behavioral discrimination between CS+ and CS– on the following day. Moreover, CS+ evoked theta oscillations in amygdala, a neurophysiological substrate for fear learning and the associated synaptic plasticity, were stronger in 5-HTTKO than WT mice. Finally, acute pharmacological blockade of the 5-HTT with citalopram during the fear conditioning sessions enhanced subsequent fear memory recall. These data are consistent with the idea that loss of 5-HTT function increases sensitivity to emotionally relevant stimuli and thereby enhances memory of those events.

### 5-HTTKO mice have superior aversive learning

Previous studies have reported normal fear learning in 5-HTTKO mice and rats^22–24,36^ but these studies used “single-cue” fear conditioning and not the discriminative approach used in the present study. Single-cue fear paradigms typically produce strong fear responses (i.e., high levels of freezing), which likely reduces the chance of detecting enhanced fear learning because of ceiling effects. Moreover, discrimination tasks require the mice to attend to the specific sensory features of the auditory cues to a greater extent than single-cue paradigms. In this respect, it is worth noting that the enhanced discrimination performance shown here in the 5-HTTKO mice was driven by responses to both the CS+ and CS– (Fig. 2), and was mirrored by larger amygdala T_O2_ responses to both the CS+ and CS– cues.

The present finding of improved aversive learning in 5-HTTKO mice complements recent studies showing impaired aversive learning in 5-HTTOE mice^25,26,37^. 5-HTTOE mice have ~3-fold greater 5-HTT expression than their WT counterparts, and lower extracellular 5-HT^18,20,25^. Our results are also consistent with data from human 5-HTT gene association studies showing superior discriminative aversive learning in putative low 5-HTT expressing individuals^38,39^, as well as a positron emission tomography (PET) study showing that individuals with lower amygdala 5-HTT expression show enhanced discriminative aversive learning^40^. Collectively, these data argue that aversive learning is influenced across a continuum of 5-HTT expression levels in humans and mice, with superior learning in low- or null-expressing 5-HTT variants.

### 5-HTTKO mice have larger fear-evoked amygdala T_O2_ responses

We found that conditioned aversive cues evoked larger amygdala T_O2_ responses in 5-HTTKO than WT mice. These findings are supported by human fMRI studies showing larger amygdala BOLD responses to emotional faces in carriers of the short allele of the 5-HTTLPR variant^5^. Our T_O2_ data are also consistent with an ex vivo auto-radiographic study, which reported a biomarker of blood-flow to be higher in the amygdala of 5-HTTKO mice following fear recall^41^. Moreover, we found that larger T_O2_ responses to the unconditioned stimulus (foot-shock) were predictive of better behavioral discrimination between the CS+ and CS–, arguing that these signals have a meaningful relationship with the observed behavioral output^27^.

Collectively these data support enhanced amygdala responses to the unconditioned and conditioned stimuli being the neural substrate for the superior aversive learning in association with loss of 5-HTT function. Moreover, we have recently shown lower fear-evoked amygdala T_O2_ responses in 5-HTTOE mice^25^, indicating that amygdala responsivity to fearful stimuli is bi-directionally modulated by changes in 5-HTT expression levels.

### 5-HTTKO mice have enhanced fear-evoked theta oscillations

We found that alongside T_O2_ responses, aversive cue-evoked theta oscillations were also enhanced in the amygdala of 5-HTTKO mice. This result is consistent with a previous study showing increased fear-evoked theta frequency synchronization between the amygdala and prefrontal cortex in 5-HTTKO mice, although this study did not report whether amygdala theta oscillations were enhanced per se^24^. Moreover, we have previously reported that fear-evoked theta oscillations are reduced in the amygdala of 5-HTTOE mice^25^, in association with impaired aversive learning^25,26,37^.

A large body of work suggests that theta oscillations are an important neurophysiological substrate for synaptic plasticity and learning in general, and for the encoding of fear memories in amygdala in particular^25,27,35^. Theta oscillations are thought to be modulated by the activity of parvalbumin (PV)-expressing inhibitory interneurons^42^, and amygdala PV interneurons are activated by cue-evoked fear^43^ and required for successful fear conditioning^44^. Recently we found that amygdala PV interneurons are activated by 5-HT in an in vitro preparation, and that this response was reduced in the amygdala of 5-HTTOE mice^43^. It is an interesting possibility that increased 5-HT sensitivity of amygdala PV interneurons underpins the increased fear learning in 5-HTTKO mice.

### Pharmacological blockade of the 5-HTT enhances fear learning

We tested whether the effects of genetic 5-HTTKO on fear learning could be phenocopied by pharmacological blockade of the 5-HTT, using the SSRI citalopram. 5-HTTKO rodents have elevated 5-HT availability, as shown using microdialysis^45^ and fast cyclic voltammetry^18^. Nevertheless, it has been argued that the negative emotionality phenotype seen in 5-HTTKO mice is of neurodevelopmental origin rather than due to elevated 5-HT availability at the time of behavioral testing^30,31,46^. Citalopram causes a marked elevation of 5-HT availability, as shown by microdialysis^47^, and here we tested whether this elevation was sufficient to affect fear learning or memory. We did not use chronic SSRI administration because this can cause widespread changes to neuronal circuitry (e.g., increasing neurogenesis, altering the expression/function of other 5-HT receptors)^48,49^, and we wanted to test the effects of increasing 5-HT availability on fear learning/memory without these potential confounds. We found that acute citalopram given before the fear learning sessions did not affect discrimination during learning but it produced significantly better discrimination during subsequent fear memory recall.

Our results are consistent with previous studies in rats^50,51^ but extends these findings by showing a specific effect of 5-HTT blockade on discrimination learning. Using a non-discriminative fear conditioning task, Burghardt and colleagues found that an acute SSRI given before training increased freezing in rats. However, they interpreted their result in terms of a short-term increase in anxiety due to SSRI administration, an interpretation that does not fit with our data for two reasons. First, we found that superior discrimination was most evident in mice that did not receive citalopram before fear memory recall and these mice showed markedly better discrimination than a group of mice that did receive citalopram before fear memory recall. Second, anxiety is often characterized by stimulus generalization, which is the tendency to conflate aversive and benign cues, treating both as if they signal threat^52^. Thus, if citalopram were to increase anxiety, it would predict impaired discrimination between the CS+ and CS–, opposite to what we observed. Our results are consistent with the hypothesis asserted by Branchi and others^16,17^ that SSRIs amplify the impact of emotionally relevant cues (and environments) that the animal or human encounters. Our data argue that SSRIs result in increased sensitivity to both aversive (e.g., CS+) and non-aversive (e.g., CS–) cues (see Supplementary Fig. S8). Indeed, our results suggest that reducing generalization between aversive and non-aversive cues or events (i.e., enhancing their discrimination), could be a mechanism through which SSRIs reduce anxiety in human patients. Thus, SSRIs may reduce anxiety by helping patients to learn to discriminate between cues that signal aversive and benign outcomes, which in turn will reduce uncertainty and therefore reduce anxiety. However, if the living environment is dominated by aversive events then SSRIs may even cause a worsening of symptoms, as recently reported by Branchi and colleagues.

### Fear learning and memory after 5-HTTKO and 5-HTT blockade

Although the present data demonstrate that both 5-HTTKO and 5-HTT blockade enhance discriminative aversive learning, this effect was seen at different time-points in the two experiments. Thus, 5-HTTKO mice showed enhanced discrimination during training but not during recall, whereas the opposite pattern was seen in citalopram-treated mice. These data demonstrate that 5-HTT blockade does not precisely phenocopy the effects of 5-HTTKO, when studied under the current conditions. This finding may reflect the consequences of lifelong loss of 5-HTT in the KO mice versus acute blockade of the 5-HTT after citalopram. Specifically, deletion of the 5-HTT throughout life may increase 5-HT availability to cause neurodevelopmental changes to key neural circuits involved in fear that increase vigilance/sensitivity to environmental cues in adulthood, as has been suggested by previous studies on the developmental origin of the 5-HTTKO phenotype^30,31^. In comparison, acute 5-HTT blockade appears to be insufficient to cause immediate behavioral changes during training, but, in combination with the aversive experience, may produce stronger learning, as evidenced by superior discrimination during the recall sessions. Further experiments combining 5-HTT blockade and aversive experiences prior to fear conditioning could test this hypothesis.

## Conclusion

In summary, here we provide evidence that both genetic and pharmacological loss of 5-HTT function increases emotional learning in a discriminative fear conditioning paradigm. The increase in fear learning observed as a consequence of genetic 5-HTTKO was accompanied by increased amygdala responsivity in the form of enhanced cue-evoked T_O2_ responses and theta oscillations. In comparison with these effects in 5-HTTKO mice, we recently reported parametrically opposite behavioral and neurophysiological effects in 5-HTTOE mice^25^.

Moreover, although the 5-HTTLPR association with negative emotionality phenotypes in humans remains highly controversial^6^, our results show that 5-HTT expression does influence fear learning and amygdala function. In this respect, our data provide important validation for human studies that report increased fear responses in low 5-HTT-expressing individuals as measured using PET ^40,53^.

An important current theory proposes that 5-HT availability does not regulate emotional state directly but rather it regulates plasticity in key neural networks underlying emotional learning and therefore influences sensitivity to emotional life events^13–15^. Thus, emotional experiences, both positive and negative, may be encoded more strongly in individuals with higher 5-HT availability. A prediction of this theory is that loss of 5-HTT expression, thereby resulting in increased synaptic 5-HT availability^18^, will facilitate aversive learning, whereas gain of 5-HTT expression will have the opposite effects. The finding of the present study and our previous work^25^ therefore provides supporting evidence for this theory.

## Supplementary information

Supplemental Material
