# Supplementary material for: Enhanced discriminative aversive learning and amygdala responsivity in 5-HT transporter mutant mice
Source: Transl Psychiatry. 2019 Apr 17;9:139. doi: 10.1038/s41398-019-0476-8 (PMC6470159; doi:10.1038/s41398-019-0476-8)
Supplement: Supplementary file 1 — Supplemental Material [file 41398_2019_476_MOESM1_ESM.docx]

**Discriminative aversive learning and amygdala responsivity is enhanced in mice with reduced serotonin transporter activity**

***Supplemental Information***

[Supplementary Methods 2](#_Toc384455)

[Supplementary Figures 10](#_Toc384456)

[Figure S1. Electrode placements in wild-type and 5-HTTKO mice 10](#_Toc384457)

[Figure S2. Pre-exposure responses in wild-type and 5-HTTKO mice 11](#_Toc384458)

[Figure S3. CS and pre-CS freezing responses in wild-type and 5-HTTKO mice 12](#_Toc384459)

[Figure S4. Locomotor activity and tissue oxygen responses to foot-shocks in wild-type and 5-HTTKO mice during first day of fear conditioning (T1). 13](#_Toc384460)

[Figure S5. Tissue oxygen data from wild-type and 5-HTTKO mice 14](#_Toc384461)

[Figure S6. CS and pre-CS freezing responses in saline and citalopram treated mice 15](#_Toc384462)

[Figure S7. Locomotor activity responses to foot-shocks in saline and citalopram treated mice 16](#_Toc384463)

[Figure S8. Freezing responses during fear memory recall 17](#_Toc384464)

[Supplemental References 18](#_Toc384465)

# Supplementary Methods

*Subjects*

Experiment 1 used 31 wild-type (WT) mice (female: n=16) and 29 5-HTTKO mice (female: n=14). Mice were generated on a 129P1 (129P1/ReJ) × C57BL/6J hybrid background [^1^](#_ENREF_1) and thereafter repeatedly backcrossed onto a C57BL/6J background for at least eight generations. Experiment 2 used 96 female C57BL/6J mice (Charles River Laboratories). Sample sizes were based on past experience and for the discrimination index behavioral measure allowed us to detect effect sizes > 0.74 (Cohen’s d) in experiment 1 and > 0.58 (Cohen’s d) in experiment 2, with 80% power and assuming α=0.05. Mice were 4-10 months (5-HTTKO and WT) or ~2 months old (C57BL/6J) at the start of testing and were housed in a temperature and humidity controlled room under a 12hr light/dark cycle (lights on 0700-1900). Testing took place during the light cycle. Mice with electrode implants were housed 2-6 per cage before surgery and singly after surgery. All other mice were housed 2-6 per cage throughout. Mice had free access to food and water throughout the experiment. The experiments were conducted in accordance with the United Kingdom Animals Scientific Procedures Act (1986) under project licenses PPL 30/2561 and 30/3068, and were approved by local ethical review for the University of Oxford.

*Surgery*

Under isoflurane anesthesia, mice (WT: n=14, female: n=8; 5-HTTKO: n=14, female: n=7) were implanted with a carbon paste electrode (CPE, gifted by Lilly UK, Surrey, UK) into the basolateral amygdala (BLA) of one hemisphere to measure tissue oxygen (T_O2_) and a silver wire electrode into the BLA of the contralateral hemisphere to measure LFPs (approximately equal numbers received left CPE / right LFP and right CPE / left LFP). CPEs were made from 200µm diameter silver wire (~270µm coated diameter, Advent Research Materials, Oxon, UK) as previously described [^2^](#_ENREF_2). LFP electrodes were made from 125µm diameter silver wire (~177µm coated diameter, Advent). Co-ordinates for BLA implantations were -1.35mm anterior/posterior, ±3.15mm medial/lateral and -5.00mm dorsal/ventral, relative to bregma. Auxiliary and reference electrodes (200µm diameter silver wire) were implanted into parietal cortex. Each electrode was soldered to a gold pin (E363/0, Plastics One, Roanoke, VA, USA), which was inserted into a pedestal plug (MS363, Plastics One) and secured with skull screws and dental cement (‘Simplex Rapid’, Associated Dental Products, Wilts, UK). Mice were allowed to recover for at least seven days after surgery.

*Tissue oxygen (T_O2_) and Local field potential (LFP) recordings*

T_O2_ was measured using constant potential amperometry, as described previously in detail ^[3-7](#_ENREF_3)^. A constant potential (-650mV relative to a reference electrode) was applied to the electrode using a low-noise potentiostat (‘Biostat,’ ACM Instruments, Cumbria, UK). The applied potential produces the electrochemical reduction of dissolved O_2_ on the surface of the electrode, such that changes in O_2_ concentration around the tip of the electrode produce directly proportional changes in the measured Faradaic current [^8^](#_ENREF_8). The spatial resolution is estimated to be a sphere with diameter approximately twice that of the electrode, i.e. 400μm [^5^](#_ENREF_5)^,^ [^9^](#_ENREF_9).

Mice were connected to the potentiostat via a 6-channel rotating commutator (SL6C, Plastics One) held on a counter-weighted arm (PHM-110P1, Med Associates) using screened cables (363-363 6TCM, Plastics One). The potential (-650 mV) was then applied to the T_O2_ electrodes for 10 minutes to ensure a stable T_O2_ signal, upon which mice were transferred to the conditioning chamber. LFPs were recorded using a differential amplifier (DP-301, Warner Instruments, CT, USA). A Powerlab® 8/30 interface (AD Instruments Ltd, Oxon, UK) was used for analogue / digital conversion and T_O2_ and LFP data were sampled continuously at 4 kHz on a Windows PC running Chart® v5 software (AD Instruments). During fear conditioning, stimulus delivery was controlled by a custom-written script in the MED-PC language that sent TTL-pulses to the computer, ensuring synchronization with the electrophysiological recordings at 1ms resolution.

*Fear conditioning*

Fear conditioning was conducted in one of two operant chambers (ENV-307A, Med Associates Inc., Lafayette, IN, USA), each with distinct visual and olfactory cues. Mice (n=60; including n=28 who had electrode implantation surgery) underwent a pre-exposure day, three training days (T1, T2, T3), and a fear memory recall day (FMR).Training was performed in one context (e.g. context A) and recall was performed in a different context (e.g. context B if trained in context A). Contexts were counterbalanced across mice. Pre-exposure was performed in a third context (context C) and was the same for all mice

Mice were trained to discriminate between two distinct auditory cues (tone, white noise), with one cue (e.g. CS+ = tone) always paired with foot-shock during the training sessions and the other cue never paired with shock (e.g. CS- = white noise). Allocation of tone and white noise to the CS+ / CS- was fully and randomly counterbalanced across mice. At the start of each training or recall session, mice were brought to the testing room two at a time and placed into one of two conditioning chambers (context A or context B) and one drop of essential oil (almond for context A, lavender for context B) was placed onto the tissue lining the waste tray. Contexts were counterbalanced so that approximately equal numbers of WT and 5-HTTKO mice were trained in each context. Following a 300 s lead-in period, mice were presented with 10 auditory cues (5 × 2900 Hz tone, 5 × white noise; all 72 dB and 30 s duration) in a pseudo-randomly interleaved order with a mean inter-cue interval of 80 s (range 60-100 s), and with the same cue type never occurring more than twice consecutively within a session. On the training days, all five CS+ trials co-terminated with mild foot-shock (0.3 mA, 0.5 s). No shocks were given during the pre-exposure, recall, or extinction sessions. At the end of each session, mice were removed from the chamber, which was then cleaned and a fresh tissue placed into the waste tray. The total session length was 1380 s.

*Data Analyses*

*Behavior*

Freezing behavior was measured using a fully-automated script in NIH Image [^10^](#_ENREF_10), which compared consecutive video frames (1 Hz sampling) for pixel changes and assigned a freezing score if the % pixel change was below a set threshold calibrated for an absence of movement except for breathing. This automated system has over 80% concordance with human ratings of freezing behavior and gives a completely unbiased measure of immobility. A detailed description can be found in Richmond et al. [^11^](#_ENREF_11). Thus even though experimenters were not always blinded to group allocation at the time of testing, freezing scores were calculated in a completely unbiased way.

To analyze CS evoked freezing responses, we calculated percentage freezing in the 30 s before CS onset and subtracted this from percentage freezing during CS presentation (Figure 1D,E). Therefore each CS had its own baseline and CS evoked freezing is presented as a difference score (Δfreezing). For example, if a mouse froze for 12 s in the 30 s pre-CS period (i.e. 12/30 = 40%) and 18 s during the 30 s CS+ (i.e. 18/30 = 60%), this would yield a Δfreezing score of +20%. Conversely, lower freezing during the CS compared to the pre-CS period would yield a negative Δfreezing score (see Figure 1C,D). We also calculated a discrimination index by subtracting the CS- Δfreezing score from the CS+ Δfreezing score (Figure 1E).

*Tissue Oxygen (T_O2_) responses*

CS evoked T_O2_ responses were calculated by subtracting the mean T_O2_ signal during the 5 s before CS onset (i.e. baseline) from the T_O2_ signal during the 30 s CS presentation. Then, the 30 s signal was divided into fifteen 2 s time bins (i.e. 0-2 s, 2-4 s, 4-6 s…28-30 s) with each data point equal to the mean value during each 2 s time bin. The foot-shock (US) was 0.5s duration but we analyzed US-evoked signals in the 30s after the shock, not including the 0.5s in which the US was administered. We averaged TO2 responses over the five CS+, CS- and US trials of each session.

For the regression analysis to investigate whether T_O2_ signals could predict behavioral discrimination, we calculated the maximum T_O2_ signal (i.e. the peak value in one of the 15 time bins) during the CS+, CS- and US periods [^12^](#_ENREF_12). The regression model used these values (T_O2__CS+, T_O2__CS-, and T_O2__shock) on training days 1, 2, and 3 to predict behavioral discrimination on subsequent training days 2 and 3, and the fear memory recall session, respectively. Stepwise linear regression was used to determine the maximum variance explained with the fewest independent variables.

*Local field potentials (LFPs)*

CS evoked local field potentials (LFPs) were band-pass filtered between 1-80 Hz. We calculated power spectra during the 10s after CS onset for each trial and then averaged these spectra over the five CS+ or five CS- trials in each session. Spectra were computed in MATLAB (The Mathworks, Natick, MA, USA) using a Fast Fourier Transform (FFT) size of 2000 samples (with a Hamming window, 50% overlap) at a sampling rate of 1kHz, giving a frequency resolution of ~0.5 Hz. For statistical analysis, the power spectral density in each frequency bin (Φ_i,_) was transformed into a proportion of the total power between 1 and 40 Hz:

$P_{i}=\left(\frac{\Phi_{i}}{\mathop{\Sigma}\limits_{1}^{40} \Phi}\right)$, where P_i_ = proportional power, Φ_i_ = raw power density (mV^2^ / Hz)

From these proportional spectra, we determined the peak power in the theta frequency range (5-10 Hz). Spectrograms (time × frequency × power) were generated in MATLAB, using a sliding time-window of 1 s, with 500 ms overlap, a frequency resolution of ~0.25 Hz, and the ‘jet’ colormap (blue = lower power; red = higher power. Spectrograms were used for visualisation but not for analysis. Note that during training day 3, the quality of the LFP recordings was poor in several mice, and so we had to remove this day from the analysis.

*Histology*

At the end of the experiments, mice implanted with electrodes were injected with sodium pentobarbitone; 200 mg ⁄ kg) and perfused transcardially with physiological saline (0.9% NaCl) followed by either 10% formol saline (10% formalin in 0.9% NaCl) or 4% paraformaldehyde (PFA). Their brains were removed and placed in 10% formol saline (or 4% PFA) for 3 days, and then transferred to a 30% sucrose–formalin solution for 24 h and frozen. Coronal sections (40 μm) were then cut on a freezing microtome and stained with cresyl violet to enable visualization of the electrodes. Only mice with electrodes in the basolateral amygdala were included in the tissue oxygen and LFP analyses (Supplementary Figure S1).

*Drug treatment*

Experiment 2 was performed in a separate group of female mice (n=96). We used female mice because during experiment 1, female 5-HTTKO mice exhibited slightly larger amygdala T_O2_ responses than males and because, in humans, depression and anxiety are more common in females. Mice were injected with saline or citalopram (10mg/kg, i.p.; Tocris Bioscience, Bristol, U.K., Cat No. 1427, Cas No. 59729-32-7) ~30 minutes before each training and/or recall session using a fully counterbalanced design (Table 1). The behavioral paradigm was almost identical to that used in Experiment 1, except with no pre-exposure session and only two days of training. We omitted the pre-exposure day and third day of training because pilot data suggested better discrimination learning using this simpler experimental design. There were four separate treatment groups: mice given saline during training and recall (Sal_Sal, n=24), mice given citalopram during training and saline during recall (Cit_Sal, n=24), mice given saline during training and citalopram during recall (Sal_Cit, n=24), and mice given citalopram during training and recall (Cit_Cit, n=24). Mice were always tested in matched pairs so that one drug-treated and one saline-treated mouse were tested at the same time and with the same cue allocations (e.g. CS+ = tone, CS- = white noise).

*Statistical procedures*

Data were analyzed using analysis-of-variance (ANOVA) or multiple linear regression in SPSS (IBM Corp., New York, USA). ANOVAs are described in the form: A_2_ × B_3_, where A is a factor with two levels and B a factor with three levels. All graphs show the mean ± 1 standard error of the mean (SEM). The familywise error was set at α = 0.05.

*Data and code availability*

All data, MATLAB code and SPSS syntax files are available upon request to the corresponding author.

# Supplementary Figures

*
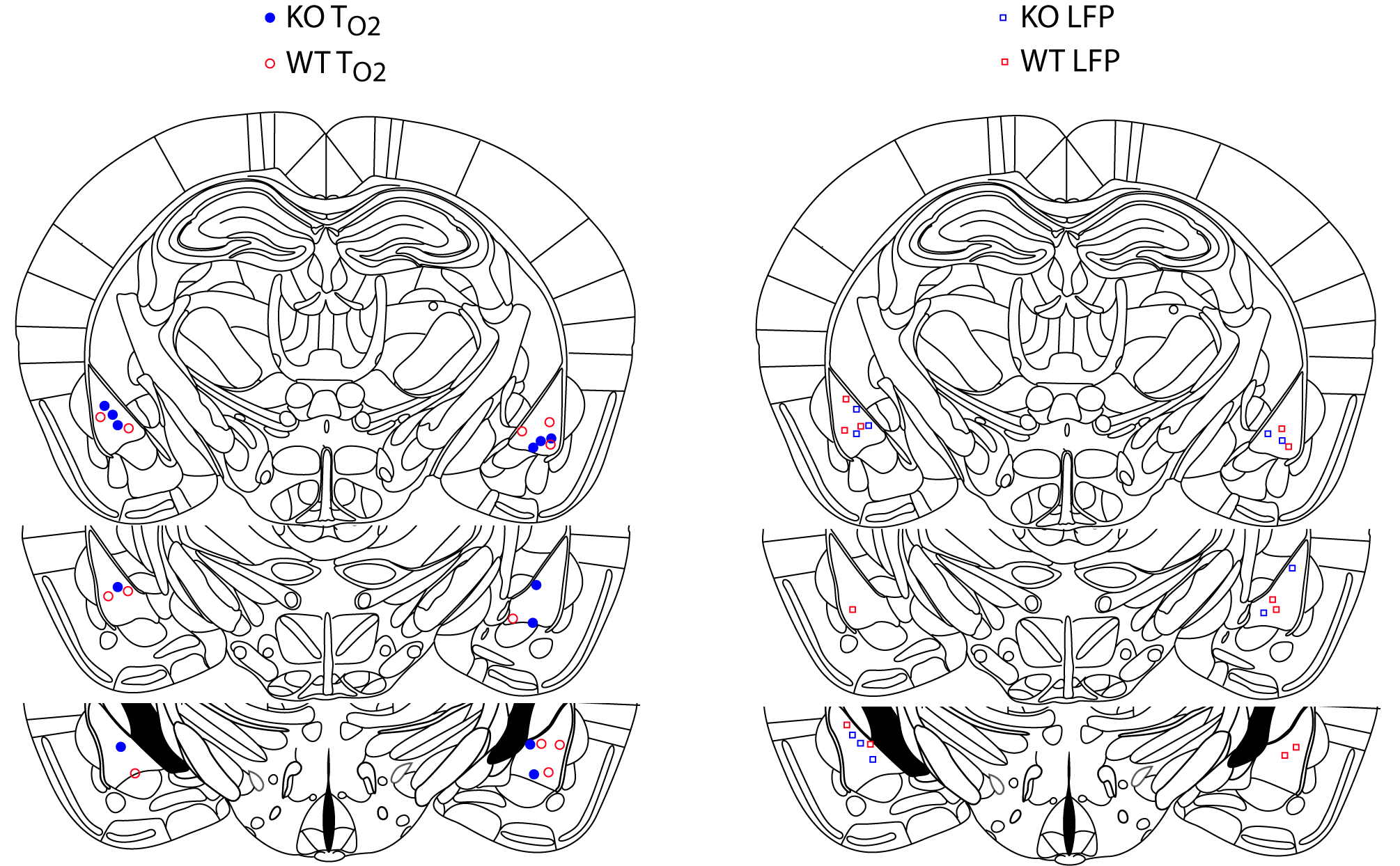
*

## Figure S1. Electrode placements in wild-type and 5-HTTKO mice

Reconstructions of basolateral amygdala electrode positions in wild-type (WT) and 5-HTT knock-out (KO) mice. T_O2_: tissue oxygen electrode (circles); LFP: local field potential electrode (squares). Figures adapted from Paxinos & Franklin [^13^](#_ENREF_13).


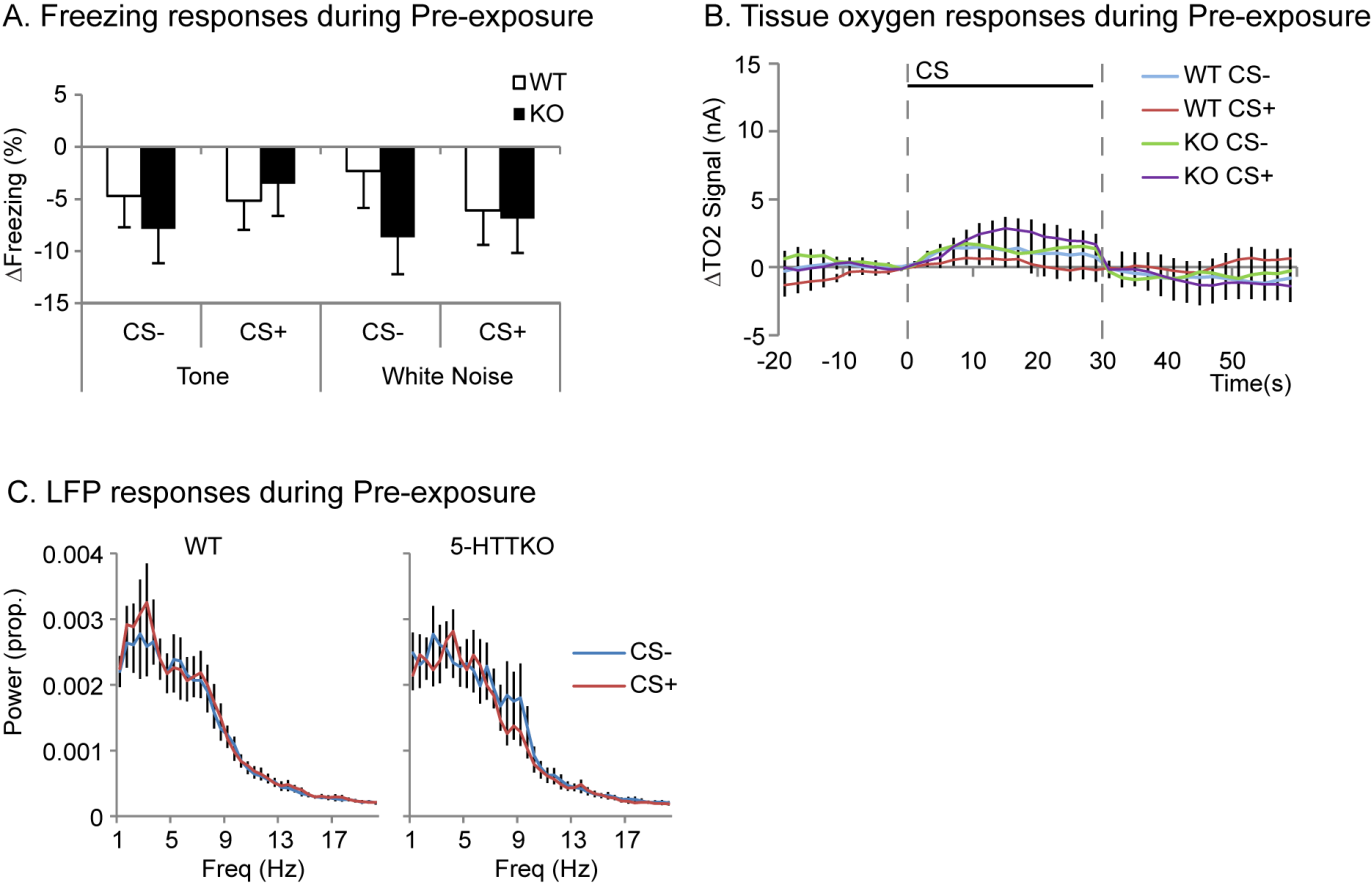


## Figure S2. Pre-exposure responses in wild-type and 5-HTTKO mice

Behavioral responses, amygdala hemodynamic responses and LFP power spectra during the pre-exposure session in WT and 5-HTTKO mice. (A) ΔFreezing responses during pre-exposure (i.e. before auditory cues were paired with shock). Cue-onset produced a slight increase in movement compared to the pre-CS period, hence negative difference scores, but there were no differences between genotypes, or between the ‘to-be-allocated’ CS types, or between the type of auditory stimulus (e.g. white noise versus tone)[all F < 1.4, all p > 0.2]. (B) Cue-onset produced a slight increase in the amygdala tissue oxygen response but there were no differences between CS types or between genotypes [all F < 1.5, all p > 0.2]. (C) LFP power spectra also showed no differences between genotypes or between CS types prior to conditioning [all F < 1, p > 0.8]. A-C show the mean ± SEM.


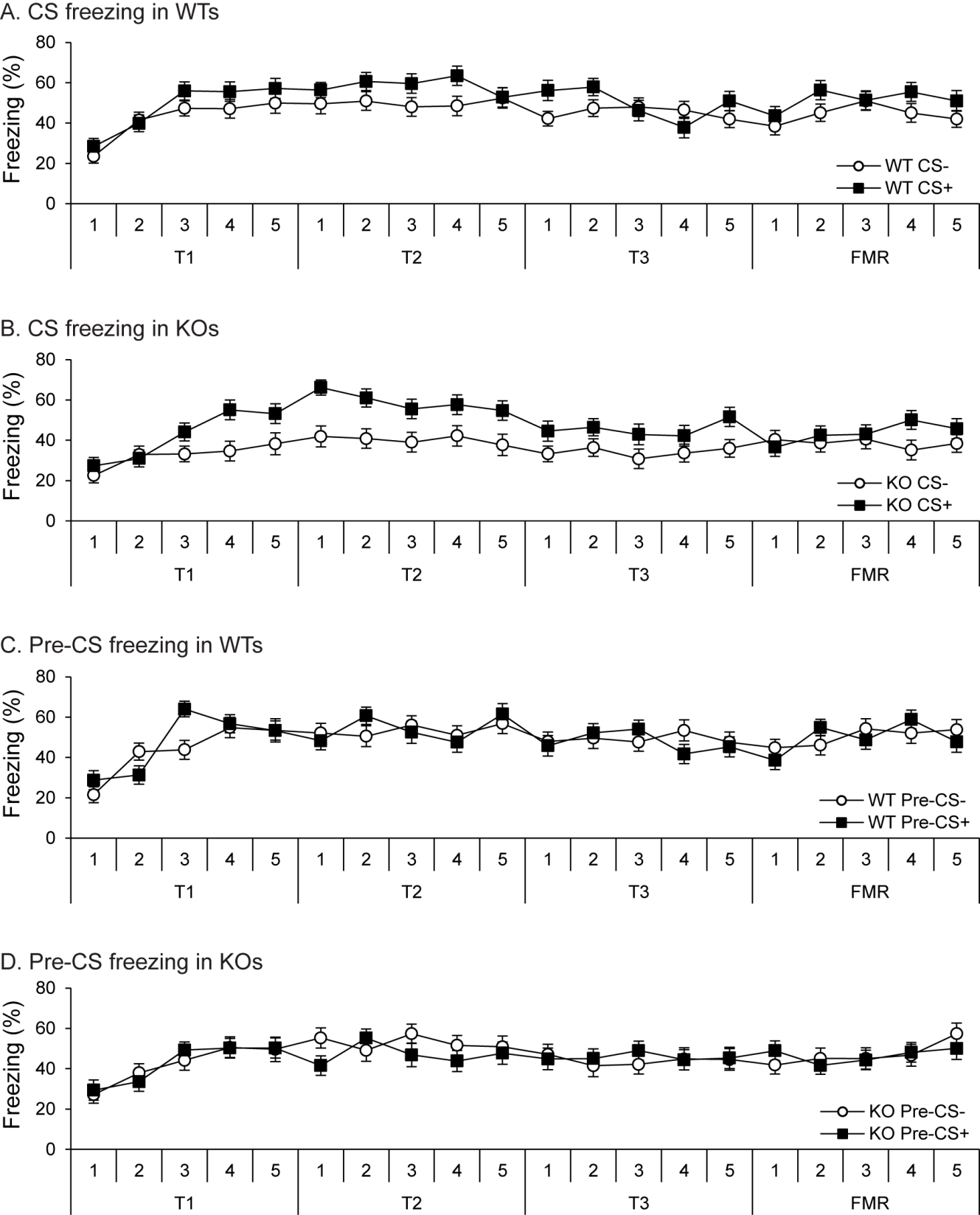


## Figure S3. CS and pre-CS freezing responses in wild-type and 5-HTTKO mice

**A-B.** Freezing during CS periods in wild-type (WT, panel A) and 5-HTTKO (KO, panel B) mice. Note that discrimination between the CS+ and CS- emerged during T1 in 5-HTTKO mice (see **B**), whereas WTs did not show significant discrimination during any trials in T1. **C-D.** Freezing levels during pre-CS periods did not differ between WT and 5-HTTKO mice (no main effect of genotype or genotype × day interaction: F < 1, p > 0.8). A-D show the mean ± SEM.


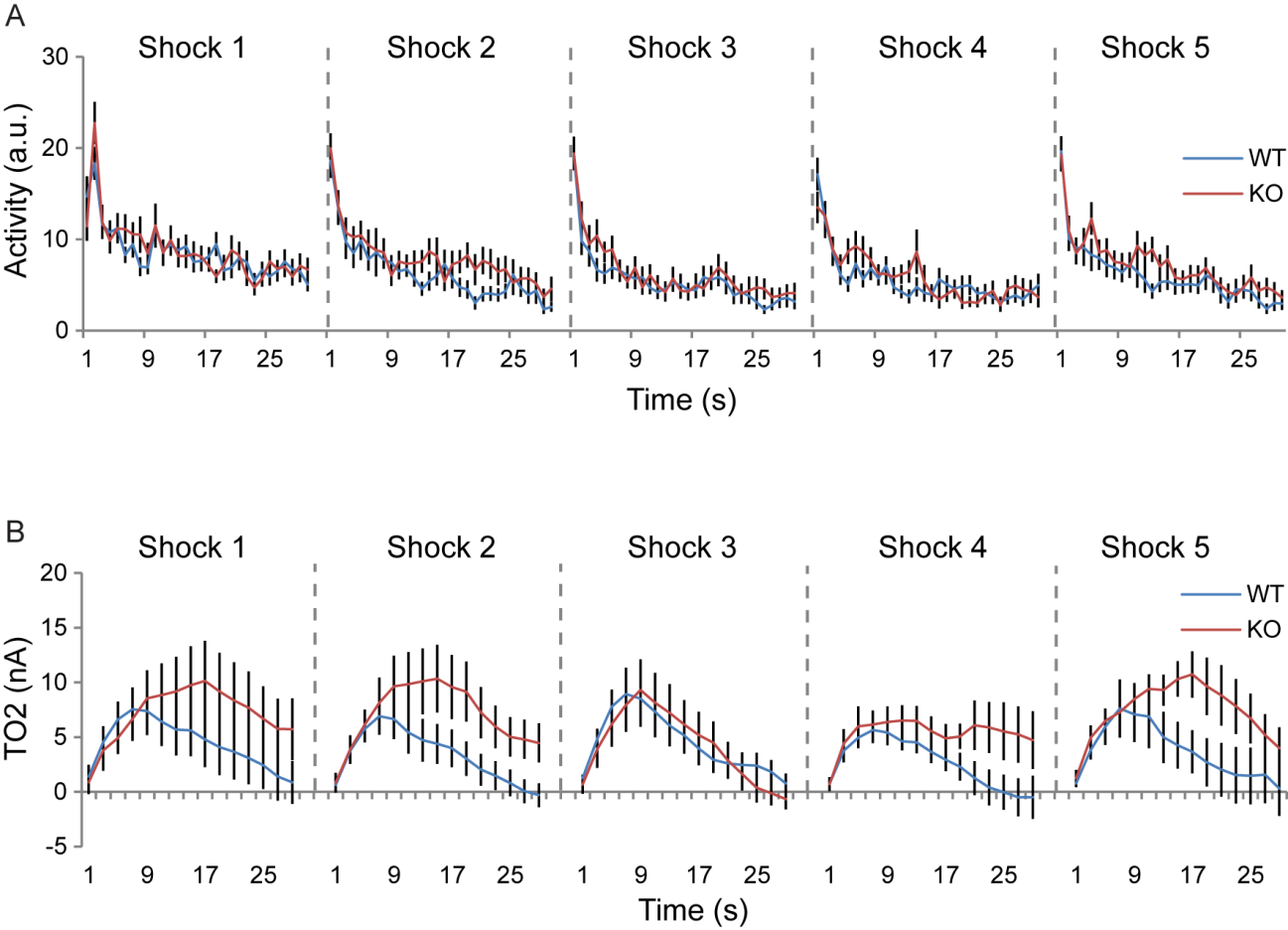


## Figure S4. Locomotor activity and tissue oxygen responses to foot-shocks in wild-type and 5-HTTKO mice during first day of fear conditioning (T1).

(A) Activity responses to the five individual foot-shocks during training day 1 (T1). Foot-shock produced a transient increase in locomotor activity but the amount of activity was comparable in WT and 5-HTTKO mice (ANOVA model: genotype_2_ × trial5 × timebin_30_, n=60). There was no main effect of genotype or interactions involving genotype: F<1.7; p>0.2). (B) In contrast, the amygdala tissue oxygen signal evoked by foot-shock was consistently higher in 5-HTTKOs vs WTs during training day 1 (ANOVA model: genotype_2_ × trial_5_ × timebin_15_ × S_24_. Genotype × timebin interaction: F(14,308)=2.1, p = 0.01). A-B show the mean ± SEM.


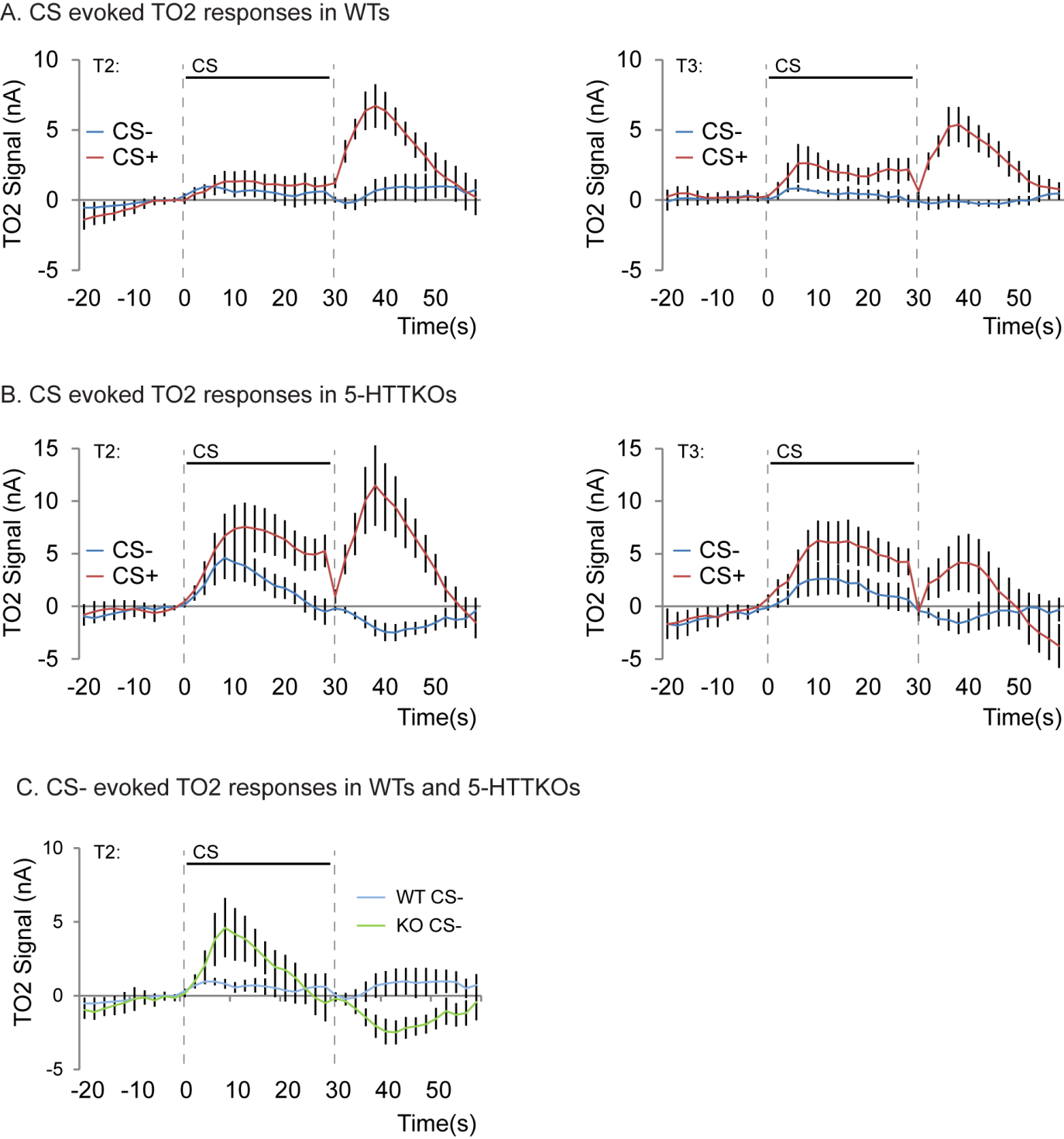


## Figure S5. Tissue oxygen data from wild-type and 5-HTTKO mice

(A) TO2 signals recorded from the basolateral amygdala (BLA) in WT mice discriminated between the CS+ and CS- by training day 3 (T3), but not during training day 2 (T2). (B) BLA TO2 signals in 5-HTTKO mice discriminated between the CS+ and CS- during both T2 and T3. (C) CS- evoked responses were larger in 5-HTTKO mice compared to WTs on training day 2 (T2). A-C show the mean ± SEM.


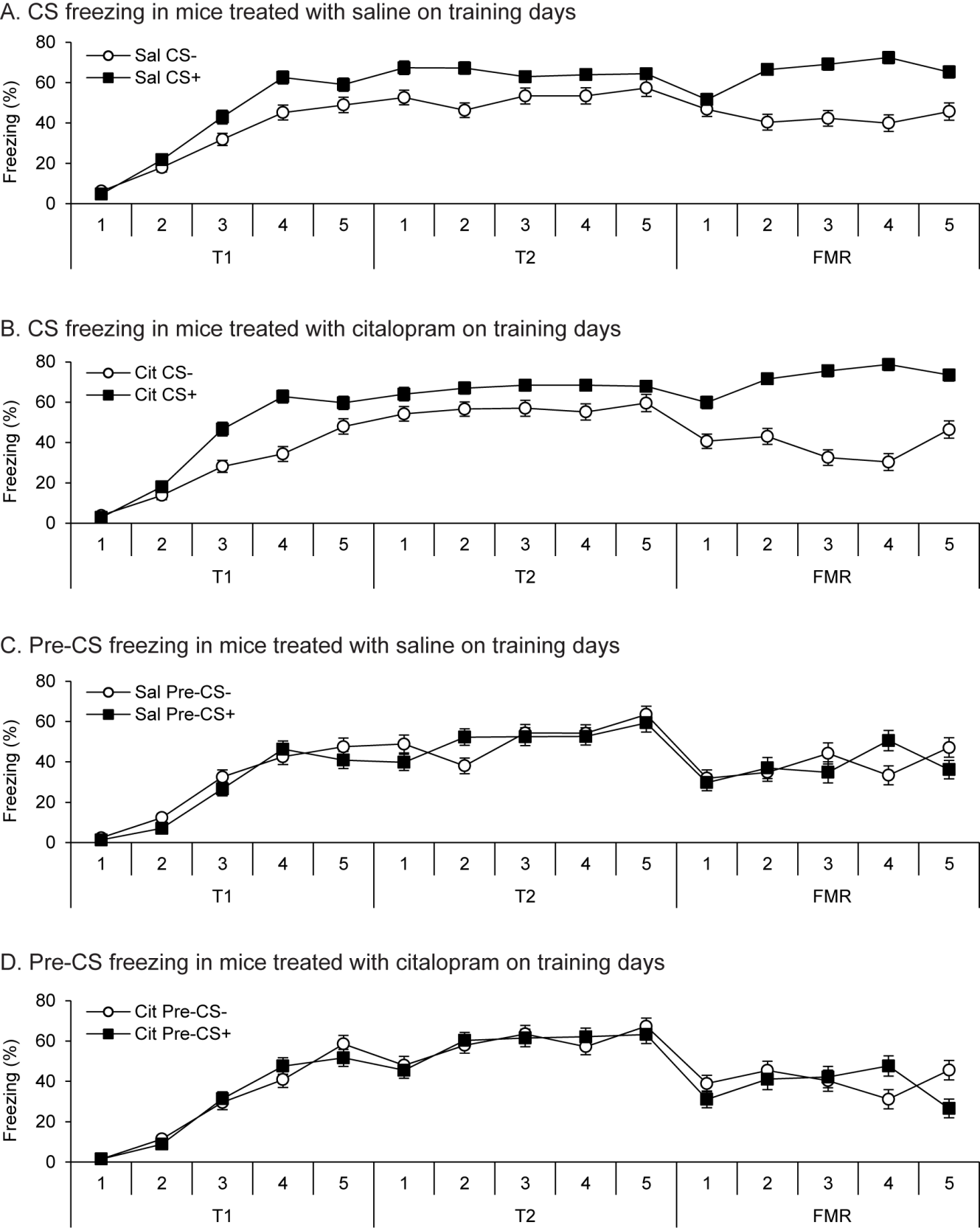


## Figure S6. CS and pre-CS freezing responses in saline and citalopram treated mice

**A-B.** Freezing during CS periods in saline (Sal, panel A) and citalopram (Cit, panel B) mice. **C-D.** Freezing levels during pre-CS periods did not differ between saline and citalopram treated mice (no main effect of drug-treatment or drug-treatment × day interaction: F < 2.4, p > 0.13). A-D show the mean ± SEM.


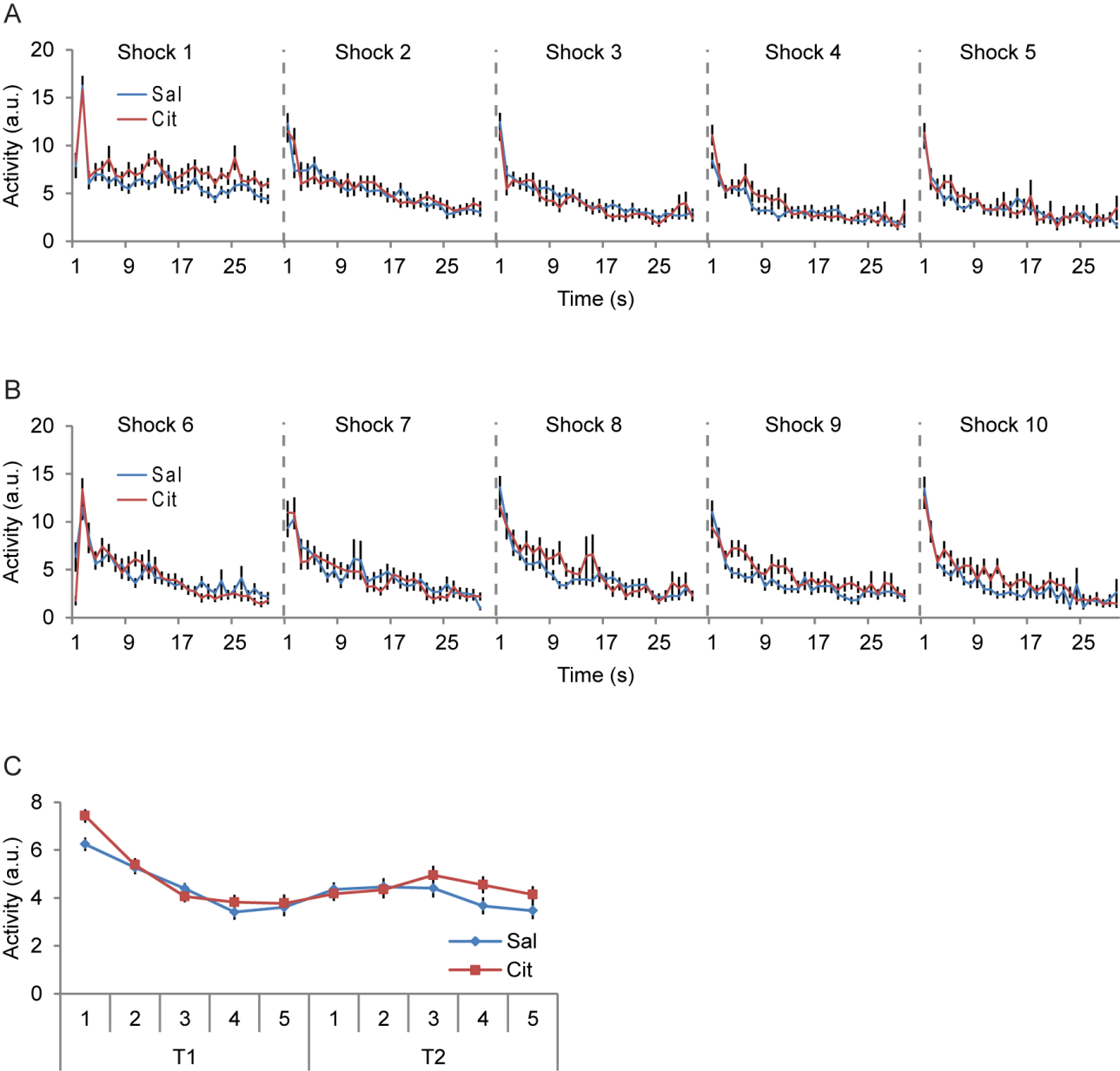


## Figure S7. Locomotor activity responses to foot-shocks in saline and citalopram treated mice

(A) Activity responses to the 5 foot-shocks during training day 1 (T1) in saline and citalopram treated mice (B) Activity responses to the 5 foot-shocks during training day 2 (T2) (C) Analyses of these data (ANOVA model: drug treatment_2_ × day_2_ × trial_5_ × timebin_30_, n=96) found an interaction between drug treatment, day, and trial (F(4,376)=3.2, p = 0.01). Simple main effects analysis found a trial × drug treatment interaction for T1 only, and pairwise comparisons revealed greater activity in the citalopram-treated mice following the very first shock (p < 0.05) but no differences in any of the other trials.


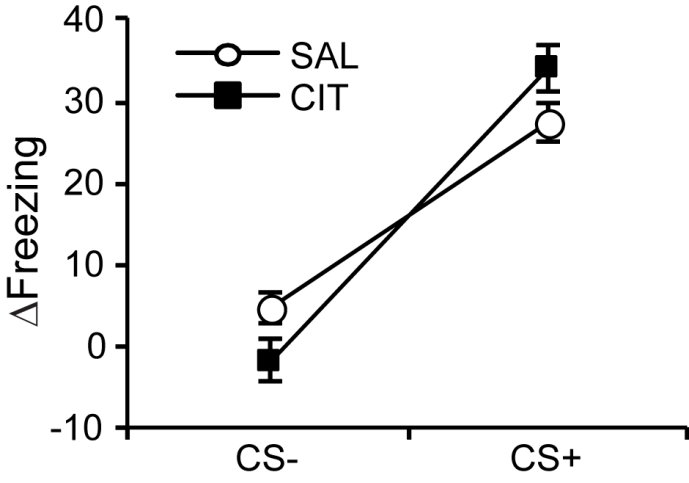


## Figure S8. Freezing responses during fear memory recall

ΔFreezing responses during CS- and CS+ trials during fear memory recall in mice that received either saline or citalopram on the training days Analyses of these data (ANOVA model: drug treatment_2_ × CS type_2_ × trial_5_, n=96) found an interaction between drug treatment, and CS type (F(1,94)=8.5, p = 0.005). Thus citalopram during training *increased* freezing to the CS+ but *decreased* freezing to the CS-.

# Supplemental References

1. Bengel D, Murphy DL, Andrews AM, Wichems CH, Feltner D, Heils A*, et al*. Altered brain serotonin homeostasis and locomotor insensitivity to 3, 4-methylenedioxymethamphetamine ("Ecstasy") in serotonin transporter-deficient mice. *Molecular pharmacology* 1998; **53**(4)**:** 649-655.

2. O'Neill RD, Grunewald RA, Fillenz M, Albery WJ. Linear sweep voltammetry with carbon paste electrodes in the rat striatum. *Neuroscience* 1982; **7**(8)**:** 1945-1954.

3. Bolger FB, McHugh SB, Bennett R, Li J, Ishiwari K, Francois J*, et al*. Characterisation of carbon paste electrodes for real-time amperometric monitoring of brain tissue oxygen. *J Neurosci Methods* 2011; **195:** 135-142.

4. McHugh SB, Fillenz M, Lowry JP, Rawlins JN, Bannerman DM. Brain tissue oxygen amperometry in behaving rats demonstrates functional dissociation of dorsal and ventral hippocampus during spatial processing and anxiety. *Eur J Neurosci* 2011; **33**(2)**:** 322-337.

5. Li J, Bravo DS, Upton AL, Gilmour G, Tricklebank MD, Fillenz M*, et al*. Close temporal coupling of neuronal activity and tissue oxygen responses in rodent whisker barrel cortex. *Eur J Neurosci* 2011; **34**(12)**:** 1983-1996.

6. Lowry JP, Griffin K, McHugh SB, Lowe AS, Tricklebank M, Sibson NR. Real-time electrochemical monitoring of brain tissue oxygen: a surrogate for functional magnetic resonance imaging in rodents. *Neuroimage* 2010; **52**(2)**:** 549-555.

7. McHugh SB, Marques-Smith A, Li J, Rawlins JN, Lowry J, Conway M*, et al*. Hemodynamic responses in amygdala and hippocampus distinguish between aversive and neutral cues during Pavlovian fear conditioning in behaving rats. *Eur J Neurosci* 2013; **37**(3)**:** 498-507.

8. Hitchman ML. *Measurement of Dissolved Oxygen*. John Wiley: New York, 1978.

9. Thompson JK, Peterson MR, Freeman RD. Single-neuron activity and tissue oxygenation in the cerebral cortex. *Science* 2003; **299**(5609)**:** 1070-1072.

10. Schneider CA, Rasband WS, Eliceiri KW. NIH Image to ImageJ: 25 years of image analysis. *Nat Methods* 2012; **9**(7)**:** 671-675.

11. Richmond MA, Murphy CA, Pouzet B, Schmid P, Rawlins JN, Feldon J. A computer controlled analysis of freezing behaviour. *J Neurosci Methods* 1998; **86**(1)**:** 91-99.

12. McHugh SB, Barkus C, Huber A, Capitao L, Lima J, Lowry JP*, et al*. Aversive prediction error signals in the amygdala. *J Neurosci* 2014; **34**(27)**:** 9024-9033.

13. Paxinos G, Franklin KBJ. *The Mouse Brain in Stereotaxic Coordinates*, 2nd edn. Academic Press: New York, 2001.
